# A Detailed Overview of Immune Escape, Antibody Escape, Partial Vaccine Escape of SARS-CoV-2 and Their Emerging Variants With Escape Mutations

**DOI:** 10.3389/fimmu.2022.801522

**Published:** 2022-02-09

**Authors:** Chiranjib Chakraborty, Ashish Ranjan Sharma, Manojit Bhattacharya, Sang-Soo Lee

**Affiliations:** ^1^ Department of Biotechnology, School of Life Science and Biotechnology, Adamas University, Kolkata, India; ^2^ Institute for Skeletal Aging and Orthopedic Surgery, Hallym University-Chuncheon Sacred Heart Hospital, Chuncheon-si, South Korea; ^3^ Department of Zoology, Fakir Mohan University, Vyasa Vihar, Balasore, India

**Keywords:** immune escape, vaccine escape, SARS-CoV-2, escape mutation, variants

## Abstract

The infective SARS-CoV-2 is more prone to immune escape. Presently, the significant variants of SARS-CoV-2 are emerging in due course of time with substantial mutations, having the immune escape property. Simultaneously, the vaccination drive against this virus is in progress worldwide. However, vaccine evasion has been noted by some of the newly emerging variants. Our review provides an overview of the emerging variants’ immune escape and vaccine escape ability. We have illustrated a broad view related to viral evolution, variants, and immune escape ability. Subsequently, different immune escape approaches of SARS-CoV-2 have been discussed. Different innate immune escape strategies adopted by the SARS-CoV-2 has been discussed like, IFN-I production dysregulation, cytokines related immune escape, immune escape associated with dendritic cell function and macrophages, natural killer cells and neutrophils related immune escape, PRRs associated immune evasion, and NLRP3 inflammasome associated immune evasion. Simultaneously we have discussed the significant mutations related to emerging variants and immune escape, such as mutations in the RBD region (N439K, L452R, E484K, N501Y, K444R) and other parts (D614G, P681R) of the S-glycoprotein. Mutations in other locations such as NSP1, NSP3, NSP6, ORF3, and ORF8 have also been discussed. Finally, we have illustrated the emerging variants’ partial vaccine (BioNTech/Pfizer mRNA/Oxford-AstraZeneca/BBIBP-CorV/ZF2001/Moderna mRNA/Johnson & Johnson vaccine) escape ability. This review will help gain in-depth knowledge related to immune escape, antibody escape, and partial vaccine escape ability of the virus and assist in controlling the current pandemic and prepare for the next.

## 1 Introduction

After detecting SARS-CoV-2 in Wuhan, the world is currently passing through a very crucial pandemic station. Infections have spread throughout the globe. Millions of people have died. School, colleges and universities are closed. The situation is a significant challenge for the world economy. It has been noted that vaccination is one of the tangible ways to fight against the pandemic. Therefore, every country has started a COVID-19 vaccination program to vaccinate the people and fight against the pandemic (1, 2). At the same time, scientists noted different new variants of SARS-CoV-2, affecting various epidemiological phenomena of the pandemic. Some significant variants have been entitled as Variants of Concern/Variants of Interest (VOC/VOI) status due to their superior risk with more severity and amplified transmissibility (3–6). These VOC/VOI possess mutations imparting properties like an immune escape and reduced vaccine efficacy to this virus (7). Due to the added devastating effects of the SARS-CoV-2 variants, Boehm et al. describe the present conditions as pandemics within the pandemic because of the spread of these variants (8).

Several significant variants of SARS-CoV-2 have emerged during one and half years due to the mutations. Some are entitled as VOC/VOI by CDC (US), and WHO, among the variants. Major VOCs are B.1.1.7 (Alpha), P.1 (Gamma), B.1.427/B.1.429 (Epsilon), B.1.351 (Beta), B.1.617.2 (Delta), which contains different significant mutations in the S-glycoprotein. Few mutations include K417T/N, E484K, L452R, N501Y, P681R, D614G, etc. (3, 9). At the same time, some significant VOIs are circulating in different parts of the world, which are B.1.526, B.1.525, P.2, P.3, B.1.617.1 (3, 10). Several mutations have been observed among VOI, and some common mutations are K417T/N, E484K, L452R, N501Y, P681R and D614G. However, most of the mutations were noted as deleterious mutations in genomes sampled from the mutant variety of the SARS-CoV-2, circulating throughout the world (5, 11). It has been observed that several characteristics of the virus may have been changed due to these mutations. Some prominent biological functions that might have changed are infectivity, re-infectivity, pathogenicity, antigenicity, and transmissibility (3). One of the crucial mutations was the D614G mutation in the S-glycoprotein, which was noted in the early phase of the pandemic in 2020 (8, 12, 13). Kim et al. first reported this novel mutation of D614G, and they concluded that this mutation might be responsible for altered antigenicity and immunogenicity. They also stated that further detailed studies would be needed in this direction (12). At the same time, Eaaswarkhanth et al. raised a question about this mutation and a link between elevated COVID-19 mortality. They concluded that D614G substitution might be responsible for higher COVID-19 mortality (14). Similarly, several other significant mutations occur in the S-glycoprotein, which are accountable for the change of the biology of this virus. However, more researches are needed about the important mutations of these SARS-CoV-2 emergences of variants. It will help to understand the mechanism in changing the biological feature of SARS-CoV-2 due to the mutations.

All viruses can mutate, which helps the virus to evade the human immune system and cause infection and re-infection (infecting the same patient twice or more) to the human. The phenomenon is called viral escape (15, 16). This occurrence is one of the significant barriers to antiviral therapy and vaccine development. During this pandemic, the researcher has observed the emergence of SARS-CoV-2 variants rapidly through evolution. Some of these variants are associated with the appearance of immune escape. Understanding the emergence of new variants is genuinely required for controlling this virus (17).

It has been noted that the variants of SARS-CoV-2 arrived rapidly throughout the globe. Some of the early identified variants were observed in Brazil and South Africa, naturally showing the immune evasion characteristics (18). Now, SARS-CoV-2 mutants are dominant strains in several regions of the world. Some mutations of these variants are related to immune escape or partial vaccine escape of SARS-CoV-2. The two mutations are noted in the RBD (receptor-binding-domain) related to immune escape. These mutations are K417N/T and E484K (19). Another mutation in the S-glycoprotein region is D614G, which is associated with immune escape (20). At the same time, vaccine escape is another problem due to the ongoing development of the new SARS-CoV-2 variants. Thus, more immunological studies are required to understand the immune escape mechanism and partial vaccine escape mechanism of these variants of SARS-CoV-2.

This manuscript provides a detailed overview of immune and partial vaccine escape of the emerging variants with escape mutations. We first illustrated a general view related to viral evolution, viral variants, and immune escape in this direction. Next, we discussed different immune escape strategies of SARS-CoV-2, primarily innate immune escape approaches such as IFN-1 production dysregulation, cytokines related immune escape, immune escape associated with Dendritic cell (DC) function and macrophages, Natural Killer (NK) cells related immune escape, neutrophils related immune escape, pattern-recognition receptors (PRRs) associated immune evasion and NLRP3 inflammasome related associated immune evasion. Likewise, we have discussed the significant mutations in emerging variants for immune escape. In this direction, we illustrated the mutations in the RBD region (N439K, L452R, E484K, N501Y, K444R) and other regions (D614G, P681R) of the S-glycoprotein. We have also discussed mutations at different locations such as NSP1, NSP3, NSP6, ORF3, and ORF8. Finally, we discussed antibody escape and partial vaccine escape of emerging variants. Vaccine evasion of BioNTech/Pfizer mRNA vaccine, Oxford-AstraZeneca vaccine, BBIBP-CorV vaccine, ZF2001 vaccine, etc., have been highlighted. Our updated review will help researchers strategize and control the pandemic situation.

## 2 Viral Evolution, Viral Variants, and Immune Escape: Overview

Viral mutations are common in nature. This common phenomenon is a part of a virus life cycle in practicality (21). Due to elevated mutation rates, more genetic diversity develops in viruses. Deleterious mutations are noted in most cases of the virus as a part of the evolution process and natural selection, maximum being in RNA viruses (22). Similarly, natural selection might favor beneficial mutations or combinations. In contrast, the recombination process might help to retain genetic diversity (23).

On the other hand, the mutation rate is a significant criterion to understand the evolution process of the virus (24, 25). A high rate of evolution is noted in several viruses. The high mutation rate is one of the significant factors, along with generation times and population size, which attribute high evolutionary rate of viruses. However, it was observed that mutation accumulation or individual mutations might benefit or harm survival. This incidence causes genetic processes that can produce even new species or drive towards endangered species (26). It has been noted that the mutation rates in RNA viruses are more compared to DNA viruses (27). Mutation of virus creates viral variants. A high mutation rate might generate several viral variants in a limited time (25, 28). One recent example is the generation of SARS-CoV-2 variants. Pachetti et al. identified mutations in the *RdRp* gene of this virus. They have found several mutations in the *RdRp* gene and identified predominant mutations in a region-specific manner. They observed mutation in 2891, 23403, 3036, 14408, and 28881 locations in the *RdRp* gene from Europe. At the same time, some mutations, such as 17746, and 18060 are observed in North American regions isolated from SARS-CoV-2 strains (28).

Similarly, several researchers have reported the mutations of the hepatitis B virus, which might have created variants of this virus. At the same time, researchers noted that these mutations have several clinical implications (29, 30). Likewise, several mutations have been noted for the influenza virus during evolution, which has created several influenza virus variants (31, 32).

Immune escape is a phenomenon when the host immune system is incapable of responding against an infectious agent, and the process is also called immune evasion or antigenic escape (15). It has been noted that the immune escape process occurs during the evolution process of the virus and helps the virus in its survival (33). Some specific mutation helps to achieve the process of immune escape, and these mutations are called immune escape mutations (5, 15, 34, 35).

Several researchers have tried to illustrate the immune escape mechanism for various viruses (**Table 1**). Rosenberg tried to discuss the immune escape mechanism for viral hepatitis (68). Thimme et al. described the different immune escape approaches of the hepatitis C virus (69). At the same time, Lhomme et al. described the different immune escape strategies to neutralize the innate immunity of the hepatitis E virus (70). Vossen et al. represented the immune evasion of virus in the light of viral evolution. In this case, they have described the strategies to counteract the immune response into three divisions: cellular immune response, humoral immune response, and immune effector functions (71).

**Table 1:** Different approaches for immune escape by other viruses are observed from time to time.

Strategies	Virus	Remarks	References
Intervention with PRRs signaling	Vaccinia virus	A46R protein targets to multiple Toll-like-interleukin-1 receptor adaptors component	(36)
	Hepatitis C virus	NS5A protein inhibits TLR mediated signaling by combining with MYD88, Extracellular vesicles cover dsRNA of hepatitis C virus to reduce activation of TLR3	(37, 38)
	Enterovirus (EV)	Viral proteinases 3Cpro and 2Apro neutralizes the PRRs signaling pathway by targeting RIG-I and MDA5 protein.	(39, 40)
	Influenza A virus	NS1 proteins of the virus impound viral dsRNA to escape from the sensing by PRRs	(41)
	Hepatitis B virus	Escape away from the cyclic GMP-AMP (cGAMP) synthase enzyme action by the packaging of the viral genome within the capsid segment	(42)
	Ebola virus and Marburg virus	VP35 protein interact with viral dsRNA genomes to prevent viral sensing by RIG-1 and MDA-5 proteins.	(43)
	SARS-CoV	Papain-like protease antagonized the TLR7 signaling pathway by the removing of Lys63-linked polyubiquitination of TNF receptor-associated factors	(44)
Overcoming the physical barrier	Dengue virus, Zika virus and West Nile virus	Break the skin barrier via permissive cells infection	(45)
	Adenovirus, swine vesicular disease virus, reovirus, Coxsackie virus	Pass through mucosa by targeting the apical junctional proteins complex	(46)
	Simian immunodeficiency virus and human immunodeficiency virus	Enter the physical barrier in numerous ways	(47)
Transcriptional factors (IRF3/7, NF-kB, and AP1) inhibition	SARS-CoV	M protein of virus stops the activation of IRF3/7 by targeting TBK1/IKK+	(48)
	Human papilloma virus 16	E6 oncoprotein from virus binds to IRF3, which inhibits self-transcriptional activity	(49)
	Ebola virus	VP35 protein stops IRF3 phosphorylation and later dimerization	(50)
	Enterovirus	Viral 3C proteases cut the IRF7 protein	(51)
	Vaccinia virus	Viral proteins A46, A49, A52, inhibit the NF-kB activation by various mechanisms.	(52)
	Human papillomavirus	Ubiquitination of IRF-3 upstream and NFkB by upregulating the UCHL1 protein by cellular deubiquitinase	(49)
	Influenza A viruses	NS1 protein prevents the nuclear translocation of NF-kB and IRFs	(53)
	MERS-CoV	ORF8b encoded protein suppresses TBK1 and MDA5 regulated NF-kB signaling and M protein stop the TBK1-dependent phosphorylation event of IRF3	(54, 55)
	SARS-CoV-2	Inactivation of TRAF3 and stop the subsequent activation of IRF3/7 and NF-kB protein	(56)
Antagonizing of Interferon-stimulated gene	HIV-2	Antagonize the tetherin protein which interacting with the rod envelope glycoprotein of virus	(57)
	MERS-CoV	NS4b proteins responsible for the enzymatic degradation of OAS-RNase L protein element	(58)
	Hepatitis C virus, Influenza A virus, Vaccinia virus	Viral proteins (NS1E2/NS5A, Tat, and E3l/K3L) particular viruses interact with PKR (protein kinase R)	(59)
Intervention of JAK-STAT signaling	Porcine reproductive and respiratory syndrome virus	Nsp11 protein bind with IRF9, and formation of the transcription factor complex IFN-stimulated gene factor 3 (ISGF3) for nuclear translocation	(60)
	Nipah and Hendra virus	Nucleoproteins prevent the nuclear accumulation of STAT1 and STAT2 proteins and inhibit with their complex formation	(61)
	Parainfluenza virus type 1	C protein interacts and keeps STAT1 proteins in perinuclear aggregates at the terminal endosome	(62)
	Rotavirus	NSP1 protein inhibits the activation of STAT1 protein	(63)
	Mumps virus	V protein stimulate the degradation of STAT-1 and STAT-3 proteins	(64)
	Herpes Simplex Virus	Inhibition of JAK-STAT signaling pathway by inducing SOCS3 protein	(65)
	Zika virus	The viral NS2B3 protein stimulates the degradation of Jak1 protein	(66)
	Human papilloma virus types 18	E6 oncoprotein of virus interacts with Tyk2 and stop the JAK-STAT activation	(67)

Due to viral fitness, it has been noted that immune escape develops in the virus. Many factors can help establish viral fitness, and one of the factors is mutational effects. Viral fitness might reduce the vaccine efficacy for some viruses through immune escape. Therefore, this area is a significant challenge nowadays because it can also describe the immune evading mechanism against vaccines (72).

Uebelhoer et al. have described that viral fitness is related to the mutations linked with the escape of cytotoxic T cells. The researchers noted two immune escape mutations, which are L1637P, and L1637S (73). Song et al. have described the role of immune escape mutations on HIV-1 fitness. They also discussed the role of escape mutation, T242N, in replication fitness and R355K mutation for early CTL escape (74). Recently, Majumdar and Niyogi described the different mutations of SARS-CoV-2 and the implications of the mutations on viral fitness (75).

## 3 SARS-CoV-2 and Different Immune Escape Strategies

After viral infection, the first line of defense mechanism tries to apply the mechanisms for viral clearance through the components of innate immunity. The component of the line of defense machinery includes a range of immune cells, a group of cytokines, interferons, and physical barriers. The immune cells include NK cells, DCs, etc. (76–78). Physical barriers for innate immunity include skin, mucosa, mucus, mucous membranes, earwax, tears, and stomach acid provide preliminary clearance. These physical barriers offer a defense mechanism against invading viruses (79, 80). The lungs are exposed to the virus frequently. Martin and Frevert describe the first line of defense mechanism in the lungs. The mucus layer, airway epithelial cells, alveolar macrophages, innate lymphoid cells, and DCs provide the first line of defense mechanism in the lungs (81). This lung defense mechanism is first exposed to the SARS-CoV-2 and other respiratory viruses such as coronaviruses (CoV), respiratory syncytial virus (RSV), influenza virus, and rhinoviruses. After crossing the defense mechanism, the pattern-recognition receptors (PRRs) try to recognize the virus components. PRRs are also a part of innate immunity (82, 83). Presently, it has been observed that three classes of PRRs are engaged to recognize virus components. These PRRs are Toll-like receptors (TLRs), a retinoic acid-inducible gene I (RIG-I)-like receptors (RLRs), and NOD-like receptors (NLRs). It has also been noted that the two receptors (TLRs and RLRs) play a significant role in the production of various cytokines and type I interferons (IFNs) during virus infection. At the same time, NLRs regulate the production of IL-1β (interleukin-1β) and its maturation process through the caspase-1 activation process (84).

### 3.1 Innate Immune Escape Approaches of SARS-CoV-2

**Figure 1:**
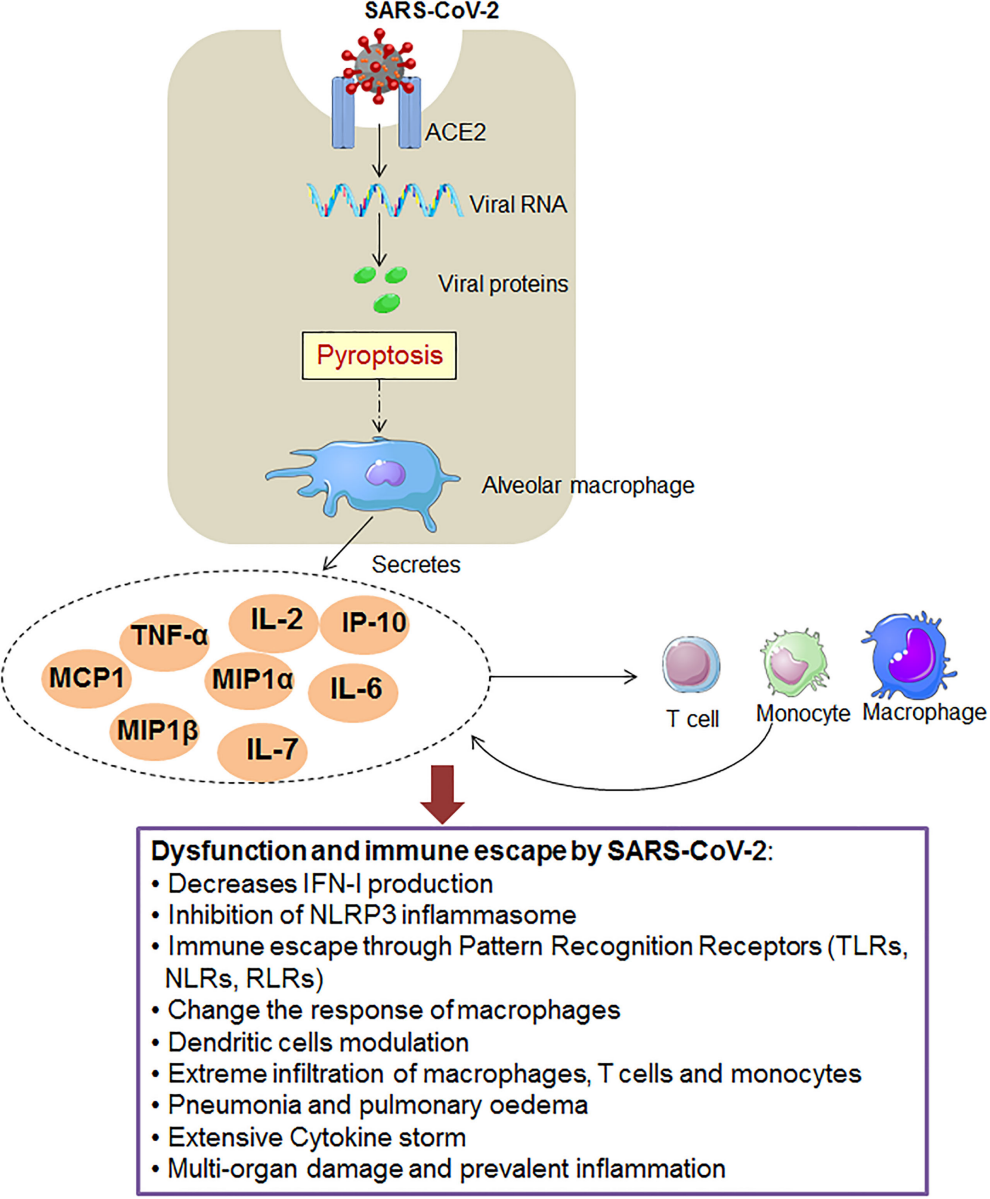
The figure shows the SARS-CoV-2 that adopts different innate immunity evasion strategies for immunity evasion.

SARS-CoV-2 adopts several innate immune escape strategies (**Figure 1**). Some innate immune escape strategies are discussed in the respective sections:

#### 3.1.1 IFN-I Production Dysregulation and Immune Escape

**Figure 2:**
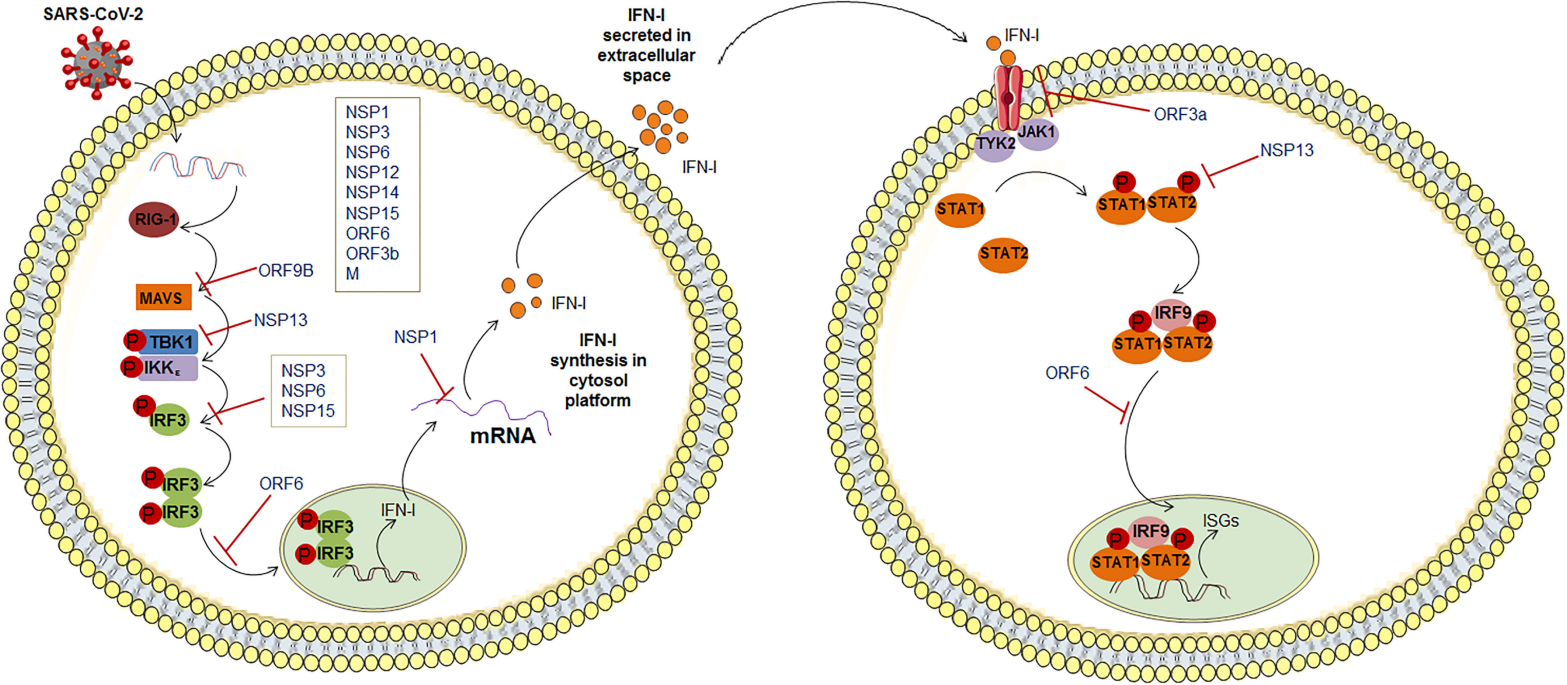
SARS-CoV-2 innate immune evasion by interfering with the IFN signaling pathway.

The SARS-CoV-2 infection causes the production dysregulation of different types of IFN (**Figure 2**). It has been observed that some kinds of IFNs production (IFN-I and -III) were reduced during infection of SARS-CoV-2 (85). It has been illustrated that type III IFN has a significant role in controlling the SARS-CoV-2. Stanifer et al. described that type III IFN might hinder the SARS-CoV-2 life cycle, thereby preventing the infection of this virus in hIECs (Human Intestinal Epithelial Cells). Therefore, interferon might be efficient in controlling the SARS-CoV-2 replication (86). However, due to the decrease or dysregulation in the IFN production, SARS-CoV-2 replication was increased. Lei et al. noted that this virus-induced an aberrant type-I IFN response in the culture cells. It might cause delayed type-I IFN responses, resulting in the evasion of type-I IFN. At the same time, S-protein and NSP2 did not show the same result (87). In contrast, Rebendenne et al. have tried to illustrate that the IFN response was incapable of controlling the replication of the virus in lung cells. However, elevated levels of IFNs produced were observed in lung cells after the infectivity of SARS-CoV-2. They reported a high level of type-I and III IFN where the IFN response was mediated through MDA-5. It has been noted that interferon production was induced to the MDA-5 associated sensing of this virus in lung epithelial cells. Thus, it might be unsuccessful in controlling the virus replication in epithelial cells in the lungs (88). Miorin et al. illustrated that this virus could inhibit STAT1 nuclear translocation, obstructing IFN signaling. They have reported that Orf6 can interact with the Nup98-Rae1 complex straightforwardly. This Orf6 protein can help in the localization of the NPC (nuclear pore complex), which suppresses interferon signaling (89). Recently, Sa Ribero et al. explained the interplay between the type-I IFN response and the virus where they have described the suppression of IFN-I induction by the virus. They represented the strategies to counteract and escape IFN-I production (90). At the same time, age-related IFN-1 dysfunction was also observed by the researchers. A sharp decline in IFN-I production was indicated due to age which might cause IFN-1 dysfunction (91, 92). We can summarize that this virus is highly susceptible to IFN-I inhibition, and it was reported by several researchers (93–95).

#### 3.1.2 Cytokines and Immune Escape

In this case, other than IFN response, other cytokines may dysregulate during the COVID-19 in patients. It has been reported that IL-6 is a major indicator of severe COVID-19 in patients (96). A study conducted with 901 patients observed elevated IL-6 in severe and ICU patients (97). IL-6 might be elevated through the TLR during the COVID-19 infection. However, the mechanism is still unclear (98–100). At the same time, it was noted that the equally pro-and anti-inflammatory cytokines are augmented in COVID-19 patients. The elevated circulatory cytokines include TNFα, G-CSF, MCP1, IL-7, IL-10, IL-2, IFN-γ, etc. (101–103). The elevated circulatory cytokines may cause immune dysregulation, and they might help the virus to evade immune responses.

#### 3.1.3 DC Function and Immune Escape

**Figure 3:**
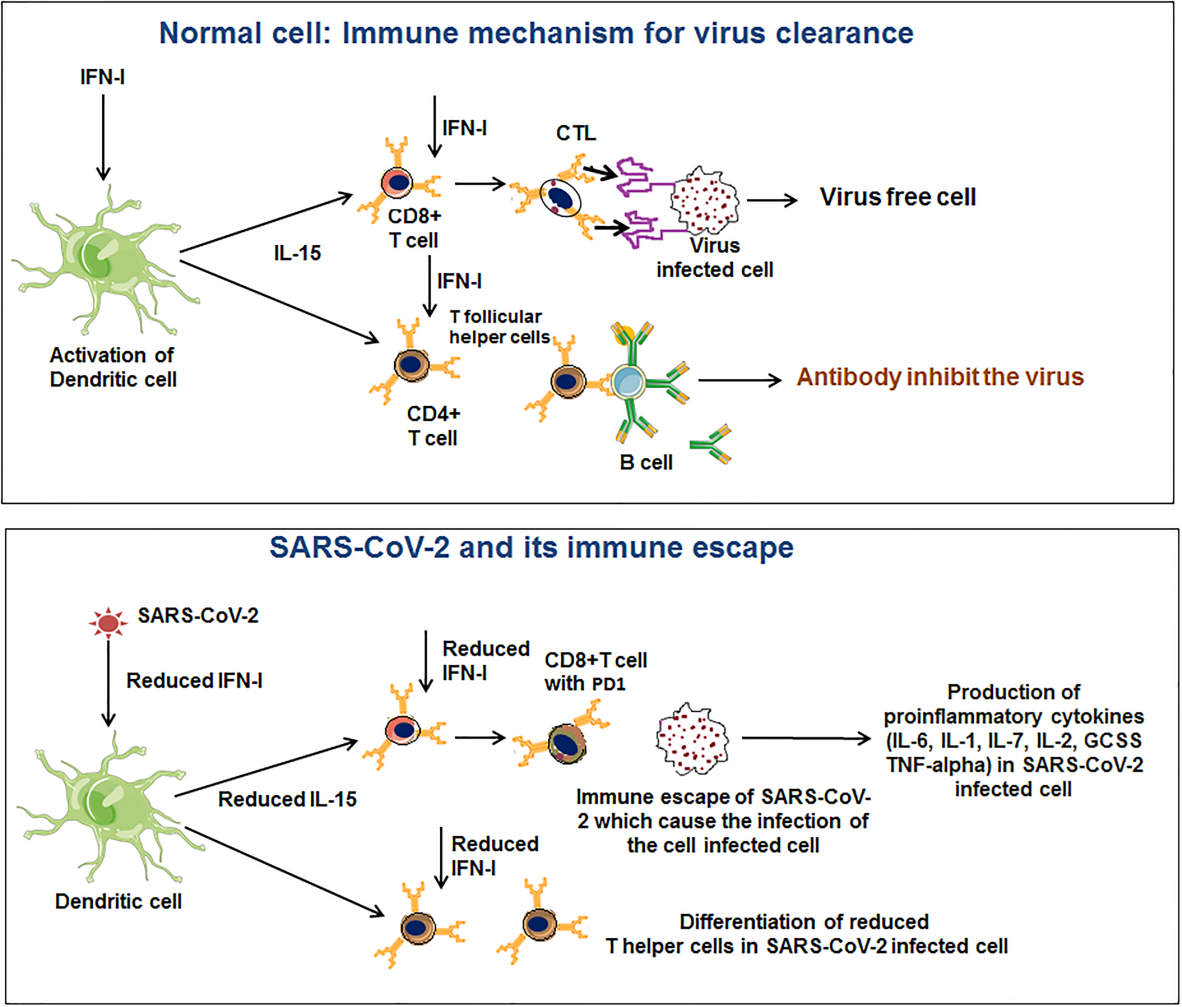
SARS-CoV-2 innate immune evasions through modulation of the dendritic cell (DC) function.

DC plays a significant role in antigen presentation and cytokine production (104, 105). DC has a major role in T cell responses (CD^8+^ and CD^4+^ T cell population) in COVID-19 patients (106–109). One recent study noted that a loss of DCs function might lead to delayed immune responses. The study indicated that SARS-CoV-2 infection causes DC and T cell responses impairment. The study has significance in the pathogenesis of the virus, extended viral transmission, disease severity, and susceptibility for future re-infection (110). It is one of the immune escape strategies adopted by SARS-CoV-2. Amino-bisphosphonates can be used to treat severe COVID-19 disease. Brufsky et al. illustrated that amino-bisphosphonates could act as DC modulators and thus can be detrimental to the ability to trigger T cells (111). Moreover, it has been observed that this virus may infect DCs and hamper the maturation of DC, restricting T cell-mediated responses (**Figure 3**) (85, 112). Therefore, DC cells have a role in COVID-19 infection progression and immune escape.

#### 3.1.4 Macrophages and Immune Escape

Another significant component of innate immunity is the macrophages. This immune cell plays an essential role during the pathogenesis of SARS-CoV. However, during SARS-CoV infection, researchers found an altered response of macrophages. Liu et al. found that s-IgG results in repeal wound healing and causes a decrease in the production process of TGF-β. This phenomenon affects the activation process of classical macrophages and the prolonged activation of classical macrophages, finally leading to severe lung injury (113). Sometimes, inflammatory macrophages accumulation is lethal to the SARS-CoV infection. Channappanavar et al. found that inflammatory macrophage with monocyte and dysregulation of IFN-I causes fatal pneumonia during the infection of SARS-CoV (114).

Similarly, macrophages can play a significant role in COVID-19 infection along with the MERS and SARS-associated coronavirus infection (115). However, macrophage subtypes play an essential role in the severity of COVID-19. Liao et al. explored the gene expression pattern in macrophage genes in a single-cell landscape in the lungs’ alveoli. They observed gene expression patterns in different macrophages such as M1-like macrophages and M2-like macrophages [alveolar macrophages (AMs)]. They concluded that the proportion of macrophage subtypes and macrophage polarization might play a pivotal role in COVID-19 severity (116).

On the other hand, SARS-CoV-2 might infect macrophages and pass through several processes to evade host immunity by manipulating macrophages (117, 118). At the same time, Dalskov et al. illustrated the immune evasion mechanism of SARS-CoV-2 by using AMs. They observed that AMs could not sense the virus (119). Again, Lv et al. illustrated that M1 type AMs help viral spread, and this cell type can uptake this virus. In this case, M1-AMs utilize cellular softness to obtain the SARS-CoV-2. Finally, the invading virus gets hold of the endolysosomal functional process to host immune escape (120).

#### 3.1.5 NK Cells and Immune Escape

NK cells are a significant part of the immune system, primarily the innate immune system, and they are one of the first lines of defense against infections against the virus. These cells help in the clearance of virus-infected cells (121, 122). On the other hand, viruses have adopted several mechanisms to escape NK cell-mediated clearance of the virus. It has been noted that several strategies have been developed by the virus to encounter NK cells. Such methods are i) targeting of NK cell receptors pathways and related ligands, ii) modulation of cytokine-mediated signaling, and iii) apoptosis (123). Such approaches are adopted by different viruses such as dengue virus, influenza virus, HIV, cytomegalovirus, etc.

Along with the other viruses, it has been noted that SARS-CoV-2 can also modulate defenses mechanism and cytotoxicity related to NK cells (124). Wang et al. found that the number of NK cells was reduced in the severe stage of SARS-CoV-2 infection. Moreover, CD^4+^ T cells were augmented in patients with COVID-19 (125). Another study by Wang et al. also showed that NK cells were decreased significantly in several COVID-19 patients (about 59% of the cases) (126). In this study, lower NK cell numbers were correlated to elevated IL-6 levels in the plasma concentrations of patients infected with the virus. NK-cell immunoglobulin-like receptors (KIR) are expressed on the surface of the NK cell, and these proteins might help evade the host responses through NK cells (127). More studies are needed in this direction to help understand NK cell-related immune escape.

#### 3.1.6 Neutrophils and Immune Escape

Neutrophils might be an indicator of viral diseases (128). In different cases, lung neutrophilia is an indicator. However, the relation between the neutrophil pool and COVID-19 is poorly understood. In COVID-19 patients, neutrophils may cause neutrophil extracellular traps (NETs) due to the diseases’ pathological effects, which cause organ damage (129, 130). Rosa et al. illustrated that neutrophil degranulation and IFN signaling are stimulated during infection of the virus. They found that neutrophil degranulation, genes associated with IFN signaling, and innate immune pathways are considerably induced in the virus-infected macaque lungs (131). At the same time, transcriptional analysis of peripheral blood mononuclear cells (PBMC) and bronchoalveolar lavage fluid (BLF) collected from COVID-19 patients showed that the four genes (LAIR1, CTSD, ADA2, GAA) are involved in neutrophil activation (132). Consequently, an augmented neutrophil-lymphocyte ratio is observed in 80% of COVID-19 patients (133–135). Therefore, neutrophil activation and accumulation may cause immune dysregulation, and viruses are proficient in escaping immune responses.

#### 3.1.7 Pattern-Recognition Receptors (PRRs) and Immune Escape

In general, pattern recognition is instigated by the communications of the genetic materials of a pathogen such as ssRNA/dsRNA/ssDNA/dsDNA, or surface proteins, such TLRs, RLRs, NLRs. The process is also associated with the COVID-19 (136). This virus modulates the PRRs for immune escape and limits IFN, other cytokines, and macrophages. Bickler et al. have evaluated that the PRR gene expression pattern of severe COVID-19 patients using genome-wide RNA-seq profiling of human dermal fibroblasts. They have found an association between age and augmented PRR gene expression (selected genes) (137). Several scientists are trying to understand the pattern-recognition of this viral protein or genomic RNA through TLR or other receptors and immune escape mechanisms using these specific groups of receptors (138–140). The virus might adopt several approaches to escape from the PRRs to immune evading. However, more studies are required to understand the complete immune evading mechanism using PRRs.

#### 3.1.8 NLRP3 Inflammasome and Immune Escape

NLRP3 inflammasome is an essential element of the innate immune system, and it activates caspase-1. In the downstream pathway, caspase-1 converts pro-IL-1β or pro-IL-18 into active forms of IL-1β or IL-18 (141–143). It has been noted that 3a protein is a significant protein of SARS-CoV-2, synthesized from the ORF3a gene. It is associated with TRAF3. This protein is related to TRAF3 through the TRAF3-binding motif. It has a role in activating the NLRP3 inflammasome and NF-κB (144, 145). After SARS-CoV-2 infection, inflammasomes are activated, and they disseminate efficiently. The massive pyroptosis induces an inflammasome activation mechanism in the epithelial cells. It causes the liberation of an enormous number of virions that cause inflammasome activation (135, 146, 147). Recently, Zheng et al. have observed that damaged NLRP3 inflammasome activation is directed to inflammatory cell death. It occurs through the RIPK3/caspase-8 pathway and might harm the host (148). At the same time, another study found that over-activation of NLRP3 inflammasome increases fatal occurrence in elderly COVID-19 patients. This study observed age-related over-activation of NLRP3 Inflammasome (149). However, Kim et al. have illustrated that NSP1 and NSP13 of SARS-CoV-2 might inhibit caspase-1 and inhibit IL-1β. This finding shows that the NSP1 and NSP13 can act as significant antagonists of NLRP3-inflammasome (150). Therefore, inhibition NLRP3 inflammasome may help the immune escape of the SARS-CoV-2.

## 4 Significant Mutations in Emerging Variants and Immune Escape

It was noted that initially, the mutation in SARS-CoV-2 was very steady. The rate of evolution was about two mutations every month, which was indicated from December 2019 to October/November 2020. The phenomenon was evident throughout the globe (151–153). After that, high mutation rates were reported, which has created several emerging variants. Presently, these variants have several significant mutations and are speeding worldwide (3). Several mutations have been found as necessary for immune escape. Some important mutations are recorded from time to time (**Table 2**) which are discussed as follows:

**Table 2:** Significat mutations are noted in different regions of the SARS-CoV-2 variants, which helps in immune escape.

Region	Protein	Mutation	Remark	Reference
Spike	Spike glycoprotein	D614G	Increase the infectivity and viral load	(154, 155)
Spike (Furin cleavage site)		P681R	Augments the viral infectivity	(156, 157)
Spike (RBD region)		N439K	Increase viral infectivity and the binding affinity to human ACE2 receptor	(158)
		L452R	Increase the infectivity and transmission ability	(159)
		Y453F	Augment the binding affinity to ACE2-receptor protein.	(160)
		E484K	Bind with monoclonal antibodies for reduction in antibody neutralization	(161)
		N501Y	Show more high transmission (cross species), binding interaction	(162, 163)
		K444R	Alteration of the virus binding affinity to ACE2 receptor	(164)
ORF1ab	NSP1	Deletions	Excess mutation and immune evasion	(165, 166)
	NSP3	Synonymous mutations	Probable impact to the fitness of the virus	(167)
	NSP6	L37F	Controls autophagy by weakening the innate immune defense system	(168)
ORF3	ORF3	Q57H, Q57H + A99V, V13L, G252V, T85I and G196V.	Cellular release of virus, change in viral function and variability	(169)
ORF8	ORF8	Deletions	Causing for milder viral infection	(170)

### 4.1 S-Glycoprotein Mutations

**Figure 4:**
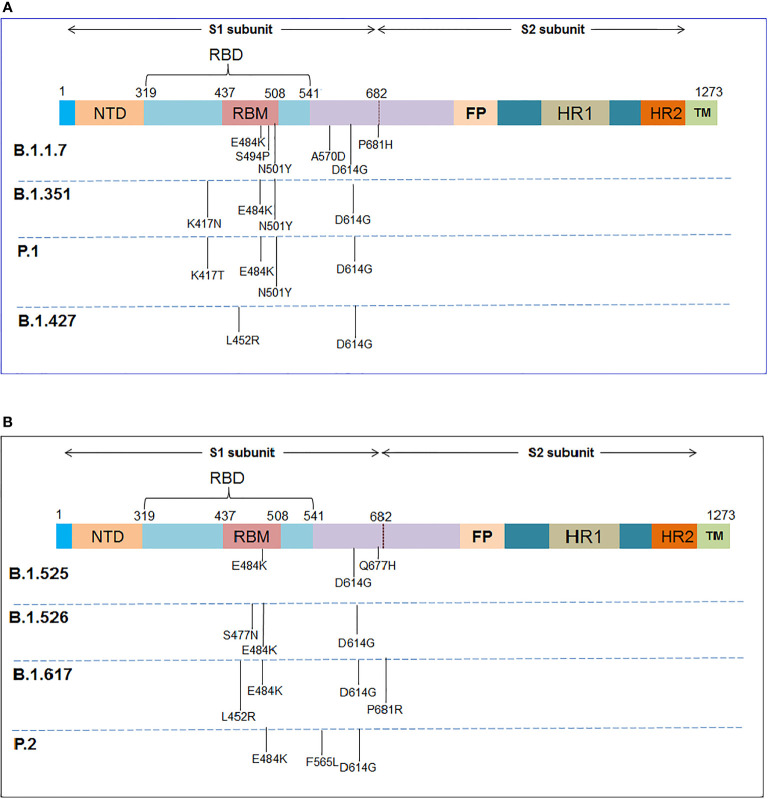
The schematic diagram illustrates the significant mutations in the S-glycoprotein as noted in the VOCs and VOIs. **(A)** Reported essential mutations in the S-glycoprotein of VOCs. **(B)** Reported critical mutations in the S-glycoprotein of VOIs.

**Figure 5:**
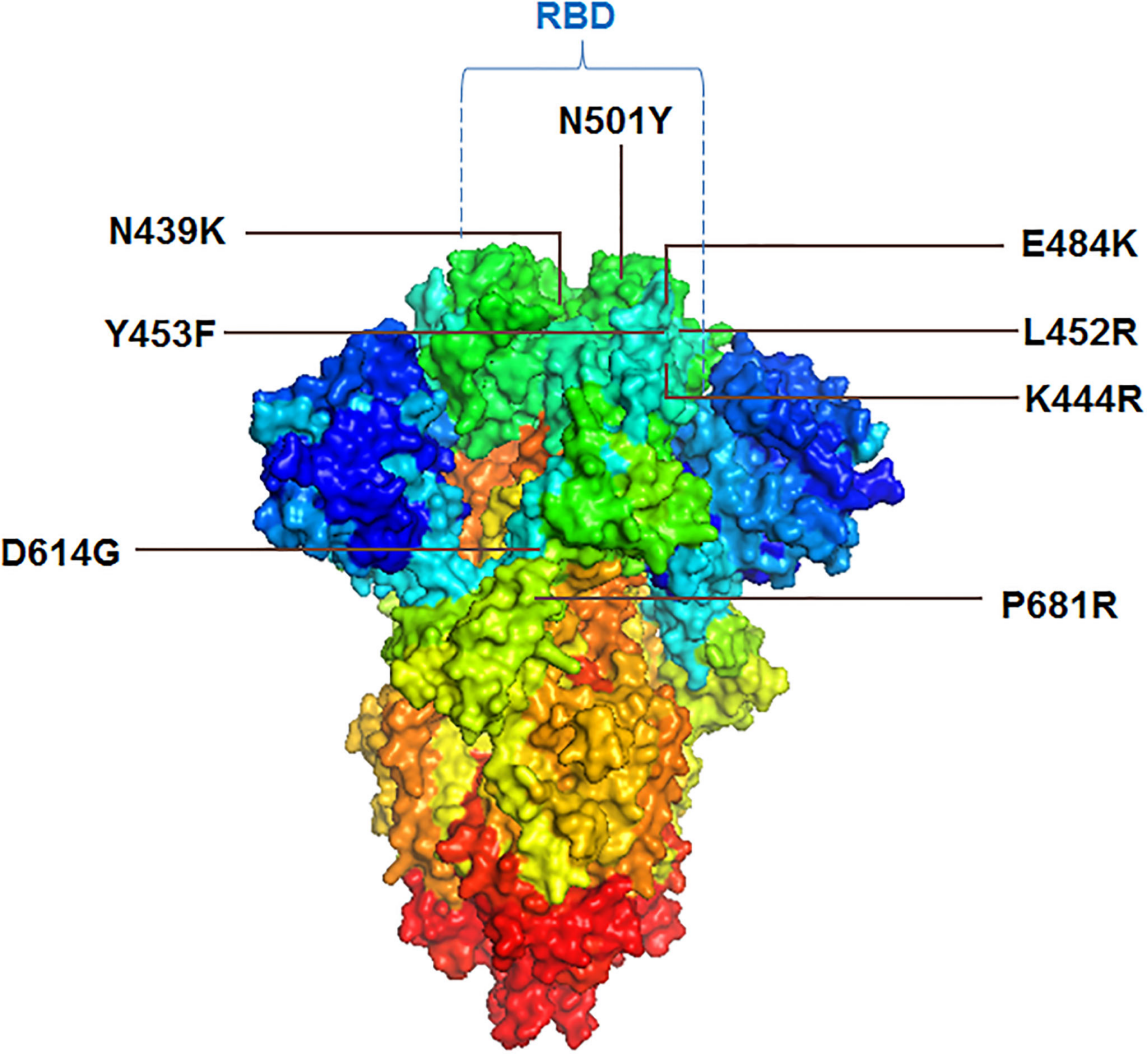
3D model of S-glycoprotein illustrating the location of significant mutations in the variants, associated with immunity evasion.

Several important mutations are reported in the S-glycoprotein region (**Figures 4**, **5**) from time to time for immune escape:

#### 4.1.1 D614G

The D614G mutation got early attention from the researchers. Researchers have noted that the frequency of this mutation started growing in April 2020. Koyama et al. reported this mutation as D614G variants. They found this mutation is frequent in different European countries, such as France, Switzerland, Netherlands, etc. (171). Scientists noted that this mutation shows higher infectivity of the COVID-19 virus (154). Several scientists pointed out that the coding sequence of the mutation (D614G) shows an augmented ratio of dN/dS. It signifies the positive selection of codon position or the mutation (172–174). Li et al. have described this mutation as highly infectious and affecting antigenicity (175). This mutation increased the affinity to ACE2-binding, leading to maximum cell entry by the virus (176). Mansbach et al. reported that this helps the open state conformation of the S-glycoprotein, which favors the virus in a higher binding rate with the host receptor. They have noted two conformational states of the S-glycoprotein concerning the S-glycoprotein mutation: D-forms (D614) and G-forms (G614). After mutation, G-forms (G614) help the S-glycoprotein’s open state and thereby help the maximum extent of interaction with the receptor (155). Daniloski et al. found that this mutation helps multiple cell types in humans and also found that the G-forms (G614) are more proteolytic cleavage resistant. This mechanism might further support the mutated virus to improve the transduction process (177). Due to all these reasons, this mutation is one of the few epidemiologically significant mutations that might help the virus escape the immune system (3, 5, 178).

#### 4.1.2 P681R

This mutation is present in the S-glycoprotein protein near the furin cleavage site. The mutation was observed in the Delta variant (B.1.617.2) and other sublineages of the B.1.617, such as B.1.617.1, B.1.617.3. For the first time, it was noted in variants isolated from India (156, 157). This mutation was also reported in Bangladesh (179). The mutation augments infectivity and may help in the immune escape phenomena of the virus.

#### 4.1.3 Mutation in RBD Region

##### 4.1.3.1 N439K

The N439K mutation is also noteworthy in S-glycoprotein. The frequency of this mutation was found to increase in some places of Europe in the first quarter of 2020. This mutation occurred due to the amino acid substitution and was found circulating in European countries such as the United Kingdom, Denmark, Germany, Sweden, Switzerland, France, Italy, Iceland, Luxembourg, Norway, and Poland. This mutation is also reported in Asian countries such as Singapore and the Middle East, such as UAE (180–182). This mutation is found in the RBM (receptor-binding motif) and increases the binding affinity to the ACE2 receptor. It is found that this mutated RBD region favor bonding with the hACE2 receptor with an augmented affinity compared to wild-type (158). Therefore, it is an epidemiologically significant mutation, and researchers are focusing on this mutation for further study. It was found that this mutation neutralizes the activity of several mAbs (monoclonal antibodies) (180, 181). The mutation may help the virus to evade immunity.

##### 4.1.3.2 L452R

Another significant mutation noted in the RBD region is L452R. It was reported from the variants isolated from several countries such as India (156) and Germany (183). The mutation has been found in several lineages such as Delta, Epsilon, Kappa, Iota, etc. The presence of mutation increases the virus’s infectivity and transmission ability. It was found from the antibody neutralization assays that there was a 2.0-fold decrease in neutralizing titers of post-vaccination plasma (159). Tchesnokova et al. describe that the mutation is associated with a strong positive selection and immune escape (184).

##### 4.1.3.3 Y453F

The Y453F mutation is also significant in the RBD region of S-glycoprotein. This mutation was found to augment the ACE2-binding affinity (160). The mutation was observed in a SARS-CoV-2 variant (mink-associated and called Cluster 5) (185). The mutation was found in mink (186). It was found that variants with this mutation were transmissible from humans to animals (187). Focosi and Maggi and other researchers have stated that this mutation is an immune escape mutation (5, 188, 189).

##### 4.1.3.4 E484K

Four mutants were identified at position 484, which are E484K/E484A/E484G/E484D. It was identified as an immune escape mutation. Among these four mutations, the E484K mutation is a very significant mutation among these mutations. It is noted in the important lineage, such as B.1.1.7 (190). The mutation was observed even in other lineages, which were isolated from different countries such as Japan (R.1 lineage), Germany, USA (B.1.1.345 lineage), Brazil, etc. (191–194). Researchers reported that mutation helps in the immune escape event. Researchers also noted the immune escape phenomenon during Bamlanivimab treatment of COVID-19 patients because of E484K mutation. The patients were infected with B.1.1.7 variant (195). Similarly, it was observed that this mutation is associated with the binding of neutralizing Abs, resulting in a reduction in antibody neutralization (161).

At the same time, another significant mutation is the E484D mutation among these four mutations (196).

##### 4.1.3.5 N501Y

The N501Y mutation is considered significant in the RBD region of the S-glycoprotein. It has been noted that the N501Y mutation displayed a more vital interaction between binding to the receptor, ACE2. Tian et al. illustrated additional π-cation and π-π interaction for the superior interaction or other force. They also concluded that the mutation with the variants shows more high transmission (163). This mutation also helps cross-species transmission through receptor binding (162). Researchers are working to detect the other variants of this mutation. Sandoval et al. developed a single nucleotide polymorphism assay to identify variants with this mutation (197). This mutation is associated with the process of amino-acid substitutions. Chaintoutis et al. reported that this mutation is associated with immune escape (198). Li et al. also illustrated that this mutation is related to immune escape (199).

##### 4.1.3.6 K444R

Three mutants were identified at 444 positions in the RBD region: K444R, K444Q, and K444N. These mutations were observed in the RBD regions in the emerging viruses. Ortega et al. found that these mutations can change the virus binding affinity to the ACE2 receptor (164). Mutation allows the virus to escape the immune system (166).

### 4.2 Mutations in Other Regions

**Figure 6:**
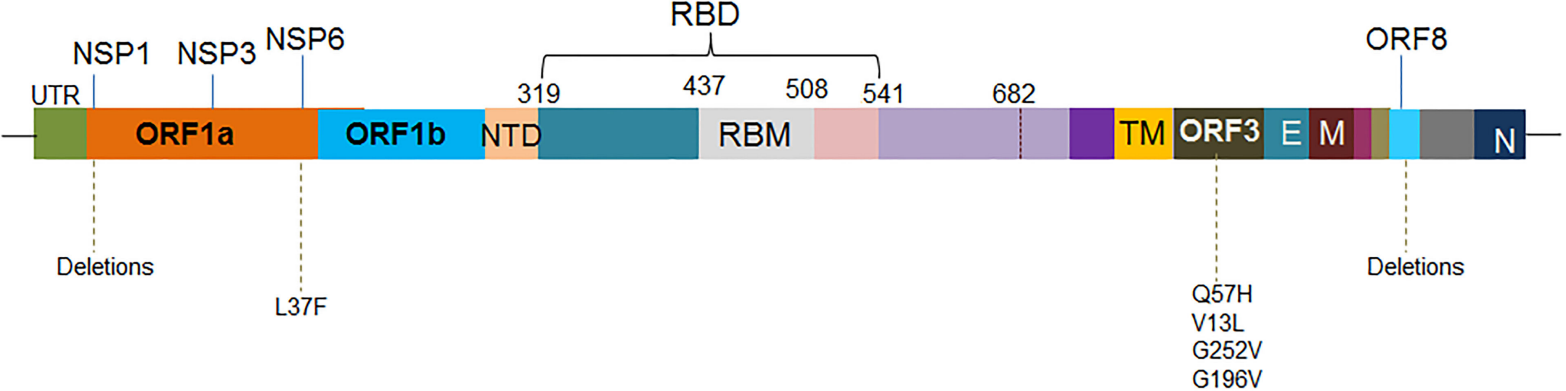
The schematic diagram illustrates the significant mutations in the other region, excluding the S-glycoprotein mutations.

Several mutations are reported in the other regions excluding S-glycoprotein mutations (**Figure 6**) which are as follows:

#### 4.2.1 Mutations in NSP1

NSP1 permits the virus in immune evasion (200). The mutation was found in different variants circulating in several countries such as Bangladesh (156). Lin et al. have found a deletion in the coding region of Nsp1 (Δ500-532). It is associated with lowering the serum IFN-β levels of virus-infected patients (201), and it may help the virus to be an immune escape.

Similarly, Benedetti et al. have observed a deletion in position 686-694 with nine nucleotides, resulting in the deletion of three amino acids (AA) to the position of 241-243 AA. However, they associate this phenomenon with decreased viral virulence (165). Therefore, all mutations are not associated with immune or partial vaccine escape in the region.

#### 4.2.2 Mutations in NSP3

Some synonymous mutations were found in this region. NSP3 of the virus is associated with pathogenesis (202), and it may influence the fitness of the virus (167). However, more study is needed to understand the role of NSP3 mutations in immune escape.

#### 4.2.3 Mutations in NSP6

Several mutations were observed in the NSP6 region. It has been noted that mutation in the NSP6 region might influence the autophagy of the virus. Therefore, this mutation is implicated in the intracellular survival of the virus and, thereby, the spreading of the virus (203). Similarly, another mutation was reported in the NSP6 at the nucleotide position, 11083, G>T mutation, which results in the mutation L37F. The mutation regulates autophagy by diminishing the innate defense mechanism against the virus (168).

#### 4.2.4 Mutations in ORF3 Region

Some mutations are noted in the ORF3 region (204). Bianchi et al. evaluated the mutations in the ORF3 region and found the five most frequent mutations, which are Q57H, Q57H + A99V, V13L, G252V, and G196V. These mutations are associated with interaction with the cellular components and, thus, change in function and variability (169). At the same time, Wang et al. found that the Q57H mutation (25563G>T) is located in the variants in the USA in high frequency. They also found that the mutation always occurs mutually with mutation 1059C>T-(T85I). Therefore, they concluded that the evolutionary trajectory in terms of time is highly alike (205). Some mutation in the ORF3 protein has been implicated in immune evasion (87).

#### 4.2.5 Mutations in ORF8 Region

Some mutations are reported in the ORF8 region. Deletion mutations have been pointed out in the ORF8 part, and Deletion of 382 nucleotides was reported in the ORF8 region. The mutation has clinical implications and might be associated with a milder infection (170). The mutations in the ORF8 region are supposed to be related to immune evasion.

## 5 Antibody Escape

**Figure 7:**
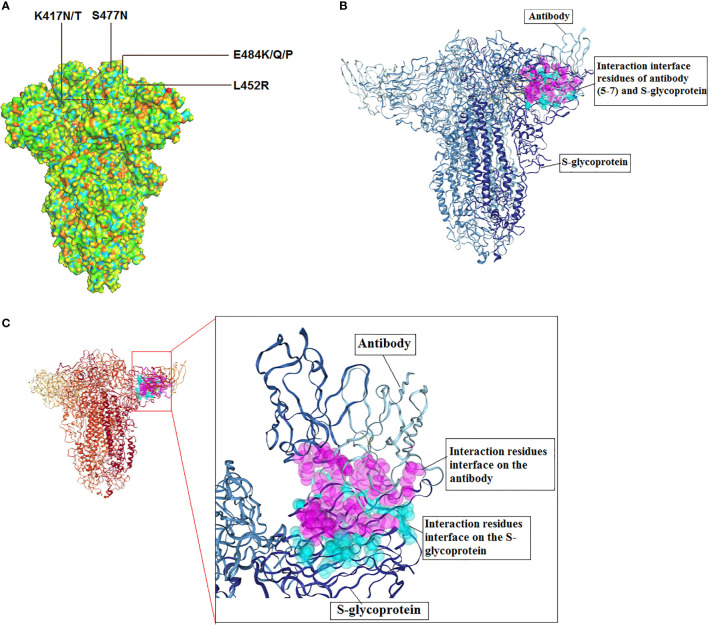
3D model of S-glycoprotein illustrating the location of significant mutations in the SARS-CoV-2, important variants related to antibody escape and model for antibody interaction. **(A)** 3D structure model shows the significant mutations for antibody escape. **(B)** A model for SARS-CoV-2 S-glycoprotein and antibody (5-7) interaction. The figure was generated using PDB id: 7RW2. **(C)** Interaction residues interface of antibody and S-glycoprotein.

Neutralizing antibodies (nAbs) are one of the significant components for adaptive immunity to neutralize different types of viruses. nAbs can be evoked through vaccination or natural infection (206). It was recently reported that the COVID-19 vaccine elicits immune responses through the nAbs production, and the nAbs may provide protection against the antigenic epitopes of the S-glycoprotein of SARS-CoV-2 (207). Different antibodies have been developed in this direction, and the antibodies are in various stages of development (**Table 3**). Several scientists reported the nAbs to escape incidents by the SARS-CoV-2 variants and their important mutations, which is a significant concern throughout the globe (**Figure 7**). Weisblum et al. reported that mutations in the NTD and RBD in S-glycoprotein of variants might confer resistance to mAb (monoclonal antibodies) from their study. Researchers have used recombinant chimeric virus (VSV/SARS-CoV-2 reporter virus) in this experiment (166).

**Table 3:** Monoclonal antibodies (mAbs) developed against SARS-CoV-2 for therapeutic purpose which are in preclinical and clinical trial stages.

Developmental stage		Sl. No.	Name and types of antibodies	Target position	Remarks
Preclinical		1.	Vh–Fc ab8, human mAb	RBD of S-protein	Bind to S-protein trimer to neutralized pseudotyped SARS-CoV-2 infections in live condition
		2.	Convalescent plasma, IgG Ab	SARS-CoV-2	Shown neutralizing activity against to the SARS-CoV-2 infection
		3.	P2C-1F11 and P2B-2F6, human mAb	RBD of S-protein	Bind with ACE2 receptor to interact with RBD, neutralizing pseudotyped and live SARS-CoV-2 infection
		4.	VIR-7831, human mAb	RBD of S-protein	Interact with the conserved epitopic part on the S-protein to neutralized the SARS-CoV-2 infection
		5.	S315, S309 and S304, human mAbs or Fabs	RBD of S-protein	Bind to the RBD, without compete RBD–ACE2 binding, also neutralizing pseudotyped and live SARS-CoV-2 infections
		6.	SAB-185, human mAb	S-protein	Neutralized the infection of live SARS-CoV-2
		7.	LY-CoV555, human mAb	S-protein	Stopped the viral attachment and entry into human host cells, neutralizing the SARS-CoV-2 infection
		8.	n3088 and n3130, human mAb	RBD of S-protein	Reduced the infections of live SARS-CoV-2 and pseudotyped
		9.	CC6.29, CC6.30 and CC12.1, human mAb	RBD of S-protein	Protect from pseudotyped and live SARS-CoV-2 infections.
		10.	4A8, 5–24, 2–17 and 4–8, human mAb	N-terminal domain of S-protein	Lowered the infections of pseudotyped and live SARS-CoV-2
Clinical	(NCT04441918)	11.	JS016, human mAb	S-protein	Targeting the S-protein to blocks the binding of virus into host cells by
	(NCT04525079)	12.	CT-P59, human mAb	RBD of S-protein	Viral load is reduced in respiratory tracts and showing therapeutic potential for COVID-19
	(NCT04454398)	13.	STI-1499, cocktail mAb	S-protein	Shown neutralizing activity against SARS-CoV-2 mutant variants ( spike D614G)
	(NCT04429529)	14.	TY027, human IgG	SARS-CoV-2	Its supports temporary protection from SARS-CoV-2 infection, used for treatment of COVID-19 patients to slow the progression of and quicken recovery.
	(NCT04483375)	15.	SCTA01, human mAb	S-protein	Competently neutralized pseudoviruses of SARS-CoV-2 by hindering the RBD of S-protein
	(NCT04592549)	16.	ADM03820, cocktail mAb	S-protein	Combination of two types human IgG1 for non-competitive binding of anti-SARS-CoV-2 antibodies
	(NCT04532294)	17.	BGB DXP593, mAb cocktails	Ectodomain trimer of S-protein	Overlapping complex structure of RBD-ACE2, used to inhibits the virus entrance
	(NCT04479631)	18.	BRII-196, human mAb	SARS-CoV-2	The epitope binding regions showed high degree of neutralizing activity against SARS-CoV-2 virus
	(NCT04561076)	19.	HLX70, human mAb	RBD of S-protein	Humanized mAbs targets to RBD used for the treatment of patients having COVID-19 and acute respiratory disorders
	(NCT04644120)	20.	ABBV-47D11, human mAb	Conserved regions of S-protein	Targets the shared epitope of viruses as cross-neutralizing antibody and potential for treatment of COVID-19 patients
	(NCT04631705)	21.	DZIF-10c, human mAb	RBD of S-protein	Protection from virus infection within the respiratory tract injected by intravenous infusion and inhalation for COVID-19 patients
	(NCT04590430)	22.	HFB30132A, recombinant mAb	S-protein	IgG4 having modified Fc shown minimized binding capability to human FcγRs
	(NCT04479644)	23.	BRII-198, human mAb	SARS-CoV-2	Shown high degree of neutralizing activity of epitope binding regions in SARS-CoV-2 virus.
	(NCT04533048)	24.	MW33, humanized IgG1κ Ab	RBD of S-protein	Recombinant antibody used for COVID-19 patients having mild or moderate infection

Similarly, Hoffmann et al. noticed therapeutic antibody escape due to the entry of the two variants (P.1 variant and B.1.351 variant). The researchers found that complete antibody escape phenomena for Bamlanivimab. At the same time, the researchers found that partial antibody escape phenomena for another therapeutic antibody (Casirivimab) (208). Another study reported the Bamlanivimab could be used to treat immunocompromised COVID-19 patients. The E484K mutation might trigger the immune escape phenomena in those immunocompromised patients (192). A recent study noted the antibody escape occurrence with combinations of antibodies. It suggested that novel spike mutants might cause loss of neutralization to the antibody cocktail, representing the escape phenomenon with combinations of antibodies. The researchers used the deep sequencing technique (209). Recently, Greaney et al. reported different mutations in the RBD might escape the binding incidence by diverse classes of antibodies. This study has noted three significant mutations (E484K, K417N/T, L452R) responsible for different categories of antibody escape. They have found that K417N/T is accountable for class 1 antibody escape. The K417N/T mutation is present in two lineages, such as P.1 lineage and B.1.351 lineage. At the same time, the E484K mutation is accountable for class 2 antibody escape. The E484K is noted in several lineages such as B.1.526 lineage, P.1 lineage, P.2 and B.1.351 lineages, etc. Similarly, L452R is accountable for class 3 antibody escape. The L452R is observed in the different lineages such as B.1.617 lineage and B.1.427/429 lineage (210).

Starr et al. prepared a comprehensive mutation map that is caused for the monoclonal antibody and cocktail monoclonal antibody escape. In this study, the researchers use the mAb LY-CoV555 and the combination of LY-CoV016 to prepare a mutation map (211). Another group of researchers has generated 50 different mutants in the S-glycoprotein region to understand the antibody escape phenomenon. In this experiment, they have used 19 mAbs the study the neutralizing incident against the mutations. They found two significant mutations (S477N, E484K) related to the antibody escape phenomenon (212). Another experiment by Starr et al. proposed breadth of the antibody might be a significant factor that can resist the antibody escape event. The study considered important mutation locations such as E484, K417, and L45 in RBM, where frequent mutations occurred in different variants (213). Other recent studies by Greaney et al. developed a wide-ranging mutation map highlighting RBD mutations. The identified mutation sites are S309, F456, E465, E484, etc. It was noted that E484 might be mutated to any of the three amino acids such as Q, K, or P. All these mutations may hinder the recognition and neutralization of the virus by polyclonal human plasma antibodies. However, they observed that some mutations (G485R and S494P) might trigger lesser antibodies escape phenomenon (214). However, several researchers tried to describe the particular interaction sties of RBD that can interact with the specific antibodies. Simultaneously, the scientists also attempted to understand the other interaction factors and significant escape mutations that may solve the problem of the fatal virus’s antibody escape property and discover the proper next-generation therapeutic antibody for the calamitous virus (215).

### 5.1 Antibody Domains Against SARS-CoV-2

Antibody domains are some naturally occurring antibodies that have a single domain. These antibodies groups are a unique class of antigen-binding fragments derived from naturally occurring antibodies. These antigen-binding fragments are also called single variable domain on a heavy chain (VHH) and are observed in the serum of camelids (216, 217). Antibody domains are the smallest antigen recognizing protein domains with a 12–15 kDa molecular weight. They are also referred as nanobodies (Nbs) (216, 217). These antibody domains are essential due to their therapeutic value. Single-domain antibodies have several advantages compared to monoclonal antibodies (mAbs). One of the significant differences is the smaller size of Nbs compared to conventional mAbs (about 150 kDa). Because of their small size, antibody domains or Nbs can access more epitopes. Simultaneously, a high amount of nanobodies (at kilogram level) can be yielded through rapid production systems such as prokaryotic expression systems. It has been reported that camelid nanobodies have revolutionized the therapy. Hence, the antibody domains are being tried to neutralize the antigenic epitopes of SARS-CoV-2. Due to the high neutralization strength, the therapeutic molecules might prevent mutational escape. Therefore, it has the possibility to neutralize a wide range of SARS-CoV-2 variants (218). Recently, Xiang et al. identified thousands of high-affinity VHH Nbs developed from the RBD-immunized llama serum. This study has chosen 109 immensely diverse sequences of Nbs to express in the *E. coli* expression system. Also, they have confirmed that 71 RBD-specific binders using an enzyme-linked immunosorbent assay (ELISA) experiment from a ninety-four purified Nbs population. Simultaneously, they found that forty-nine Nbs have a high affinity as well as high solubility. Finally, they have suggested fusing the antiviral Nbs in the remarkably steady albumin-Nb constructs, which can augment the pharmacokinetics condition (219). In another study performed by Sun et al. utilizing Antibody domains, two VH domains were selected to develop single-domain antibodies. This domain was fused to the Fc domain to augment the half-life in circulation. The two VH domains are VH m39 and VH ab6 which can be used for the therapeutic purpose against COVID-19 infection. Moreover, due to their uniqueness, these VH domains can be used for diagnosis (220). In the other work, Wu et al. developed a phage-displayed single-domain antibody library to develop single-domain antibodies (antibody domain) that can target five types of SARS-CoV-2 epitopes. In this study, they have grafted naive complementarity-determining regions (CDRs) into another region (framework region) of a human germline immunoglobulin heavy chain variable region (IGHV) allele. It has been noted that some of these single-domain neutralizing antibodies are able to neutralize SARS-CoV-2. Specifically, single domain neutralizing antibodies neutralize the SARS-CoV-2 S-glycoprotein trimeric interface of the cryptic form. These single-domain antibodies might act as a promising therapeutic for SARS-CoV-2 and might serve as anti-SARS-CoV-2 antibodies (221). Simultaneously, Koenig et al. developed four neutralizing nanobodies, which are VHHs U, V, W, and E. These nanobodies can bind RBD of S-glycoprotein of the SARS-CoV-2. By utilizing cryo-electron microscopy and X-ray crystallography, the distinct binding abilities of these antibodies toward two epitopes was demonstrated. They showed that this modular combination of Nbs can neutralize the S-glycoprotein viral variant and thus, prevent the emergence of SARS-CoV-2 variant antibody escape (222). However, more studies are needed to understand the escape phenomena and mechanisms of single-domain antibodies against the SARS-CoV-2.

## 6 Convalescent Plasma (CP) Response and Its Escape by the SARS CoV-2 Variants

For the treatment of COVID-19, convalescent plasma (CP) was considered. In this direction, several studies have been performed. Duan et al. used 200 mL of CP derived from the donors, COVID-19 recovered individuals with nAb titers higher than 1:640 and transfused them to the COVID-19 patients. The study found potentially improved clinical outcomes (223). Volk et al. have analyzed plasma-derived IVIG/SCIG products from commercial lots to understand the neutralization capacity. The study found that around 10% of products contained above 600 IU/mL neutralizing capacity. At the same time, they found that 50% of CP donations had low or no neutralizing power (224). Several clinical trials have been performed to understand the efficiency of CP to patients with COVID-19 (225). Several other studies were performed to comprehend the neutralizing capacity, nAb titer level, and other factors (226). However, recent variants of SARS-CoV-2 with the new mutations have created an alarm for the neutralizing capacity of CP to treat the disease (227). We hope future studies will develop a new technology to match the CP characteristics based on every SARS-CoV-2 variant type and infected individuals.

## 7 Partial Vaccine Escape

**Figure 8:**
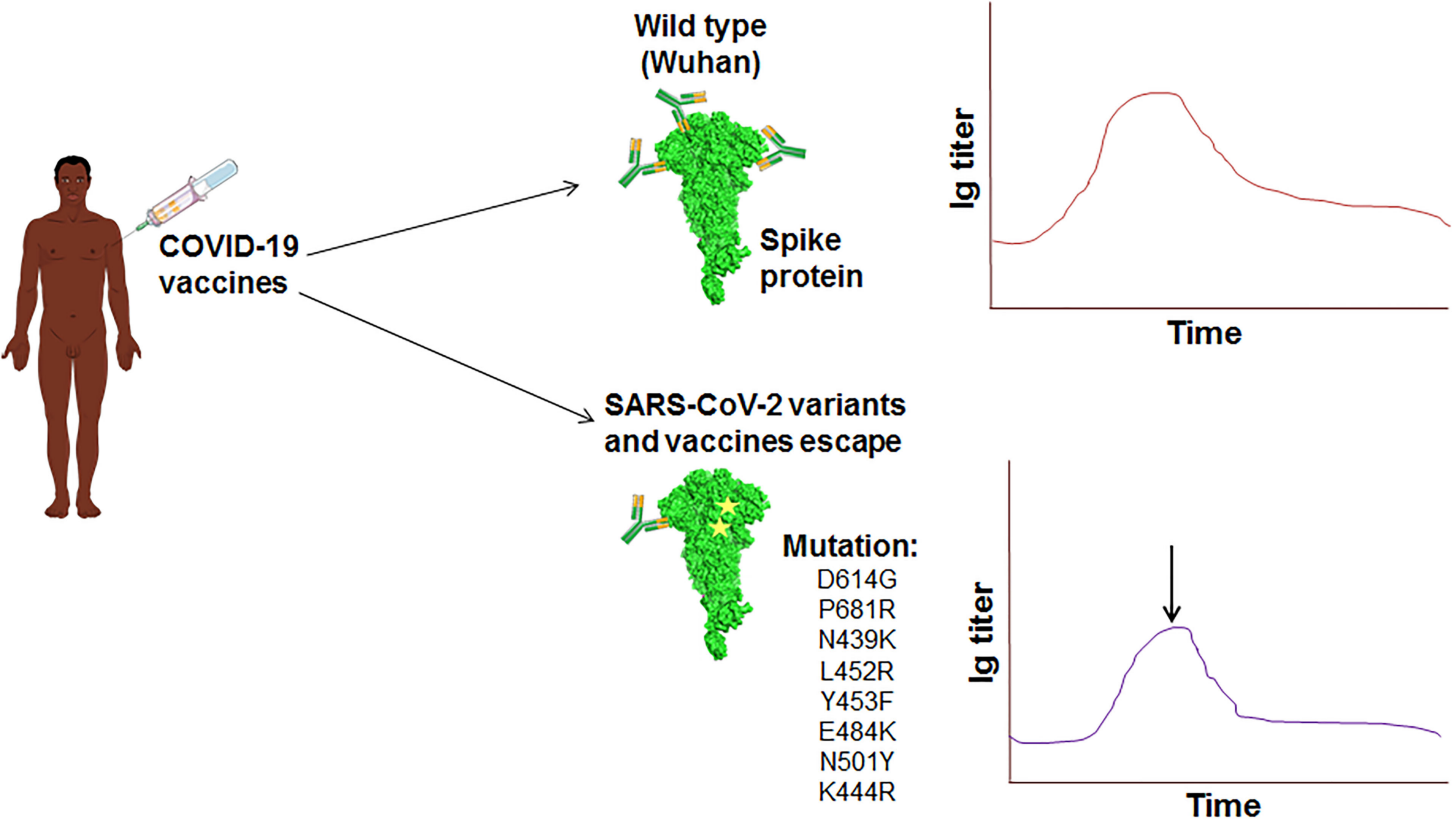
The schematic diagram illustrates the vaccine escape and indicates the vaccine escape mutations.

**Figure 9:**
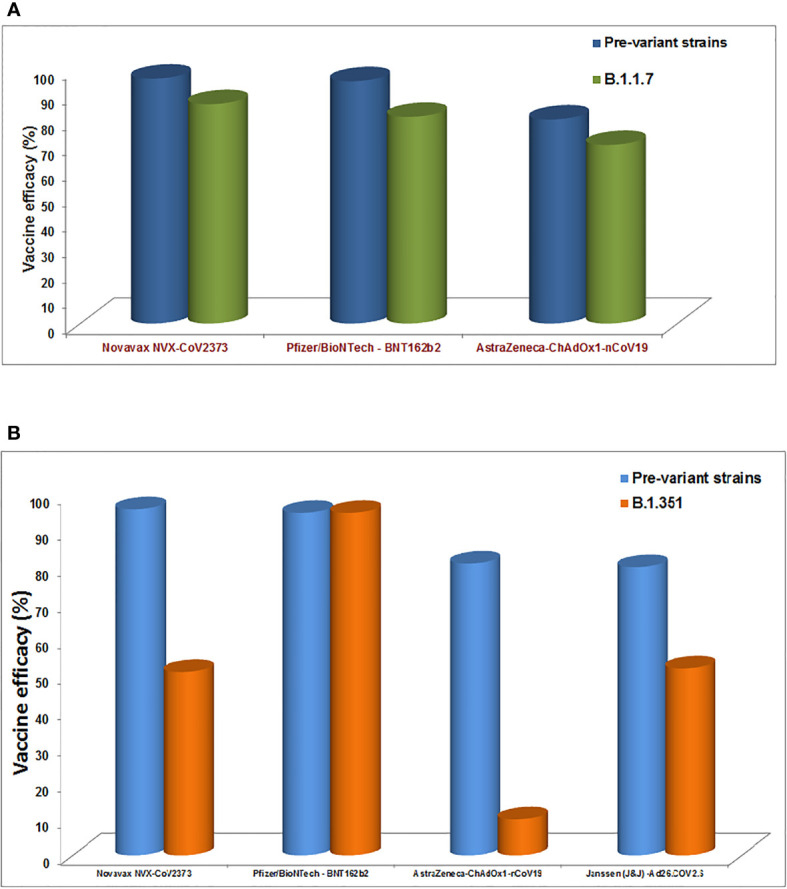
The diagram shows the impact of different vaccines effectiveness by B.1.1.7 variants and B.1.351 variant, indicating the reduction in the vaccine effectiveness. **(A)** Impact of different vaccines effectiveness by B.1.1.7 variants. **(B)** Impact of different vaccines effectiveness by B.1.351 variant.

Several COVID-19 vaccines have been licensed by several countries’ regulatory authorities and are rolled out for vaccination (**Table 4**). Recently, several studies have been performed (such as neutralization assays) to understand the vaccine efficiency related to the effect of different variants and their mutations (228, 229). It has been noted that the efficiency of vaccines is reducing in most cases due to the mutations in the variants (230). Vaccine escape is an interesting phenomenon, and therefore, it is urgently necessary to understand vaccine efficiency due to the emerging variants and their mutations **(** **Figure 8** **)**. Furthermore, there is no evidence that these partial escapes resulted from vaccine-induced antibody pressure. In this direction, assays are being developed with Pseudoviruses, which are tested with sera (postvaccination). The sera are collected from the postvaccination individuals (230). Several studies have been performed to understand the impact of different vaccine effectiveness, especially using two variants (B.1.1.7 and B.1.351 variant), which indicates a reduction in vaccine effectiveness **(** **Figure 9** **)**. We have recorded the partial vaccine escape phenomena by the different variants of the virus from time to time (**Table 5**), which are as follows:

**Table 4:** Approved COVID-19 vaccines and their developers, country of origin, efficacy, and approval month.

Sl. no	Vaccine name	Developer	Country of origin	Efficacy	Month of approval
1.	CoronaVac	Sinovac Biotech Ltd.	China	78%	April, 2021
2.	Sputnik V	Gamaleya Research Institute of Epidemiology and Microbiology	Russia	91.6%	December, 2020
3.	Ad5-nCoV	Beijing Institute of Biotechnology, CanSino Biologics	China	66%	February, 2021
4.	Janssen COVID-19 vaccine	Janssen Biotech Inc., Beth Israel Deaconess Medical Center	United States, Netherlands	66%	February, 2021
5.	ZF2001	Chinese Academy of Sciences, Anhui ZhifeiLongcom Biologic Pharmacy Co. Ltd.	China	72%	February, 2021
6.	CoviVac	Russian Academy of Sciences	Russia	–	February, 2021
7.	Oxford–AstraZeneca, COVID-19 vaccine	University of Oxford, AstraZeneca PLC	UK, Sweden	76%	December, 2020
8.	BBV152 (Covaxin)	Indian Council of Medical Research (ICMR), and Bharat Biotech Ltd.	India	81%	March, 2021
9.	EpiVacCorona	State Research Center of Virology and Biotechnology VECTOR	Russia	–	Japan, 2021
10.	BBIBP-CorV	China National Pharmaceutical Group Corporation, Beijing Institute of Biological Products, Wuhan Institute of Biological Product	China	79.3%	January, 2021
11.	Pfizer–BioNTech COVID-19 vaccine	BioNTech SE, Pfizer Inc.	Germany, United States	95%	December, 2020
12.	Moderna COVID-19 vaccine	Moderna TX, Inc., National Institute of Allergy and Infectious Diseases, Biomedical Advanced Research and Development Authority, US	United States	94%	December, 2020
13.	WIBP-CorV	Sinopharm: Wuhan Institute of Biological Products Co., Ltd	China	72.8%	February, 2021
14.	QazCovid-in	Research Institute for Biological Safety Problems	Kazakhstan	–	April, 2021
15.	Minhai COVID-19 vaccine	Minhai Biotechnology Co. and Kangtai Biological Products Co. Ltd	China		May, 2021

**Table 5:** Vaccine efficacy against the SARS-CoV-2 significant variants.

Sl. No.	Variants	Vaccine and efficacy				Reference
		Novavax NVX-CoV2373	Pfizer/BioNTech - BNT162b2	AstraZeneca-ChAdOx1-nCoV19	Janssen (J&J) -Ad26.COV2.S	
1.	B.1.1.7	The vaccine efficacy was 96% against the pre-variant strains, lowering to 86% in occurrence of B.1.1.7	Efficacy was 95%, in occurrence of B.1.1.7 the efficacy recorded 81.5%.	Earlier the vaccine efficacy was noted 81%, reduced as 70% against B.1.1.7	–	(231, 232)
2.	B.1.351	Efficacy reduced from 96 % to 51%	100% operative	Efficacy observed 10% only	52% efficacy observed against the moderate infection, and for severe disease (64% South Africa) (72% efficacy USA)	(8, 232, 233)
3.	P.1	–	Efficacy reduced from 95 % to 6.7%	–	–	(19)

The data shows the reduction or neutralization of post vaccination serum and the reduction impact of vaccine effectiveness or the partial vaccine escape.

### 7.1 BioNTech/Pfizer mRNA Vaccine

Recently, a pseudovirus of B.1.1.7 variants was developed in a study to understand the efficiency of the mRNA BNT162b2 vaccine. The study found a reduction of immune sera with a small amount. The study concluded that this variant might not escape the vaccine (BNT162b2) (234). Wang et al. have performed an analysis using a cohort of 20 human volunteers to understand the effect of variants on mRNA vaccine of Pfizer-BioNTech (BNT162b2) or Moderna (mRNA-1273). Here, the cohort received these mRNA vaccines. Using pseudotyped reporter virus, they constructed the virus with mutations, E484K/K417N/N501Y. The researchers observed the vaccine escape phenomenon using the pseudotype neutralization assay. They concluded that mRNA vaccines’ potential loss of efficacy might need to be updated occasionally to understand the effects of variants on mRNA vaccines (235). At the same time, some studies were conducted using live viruses to understand the efficacy of mRNA vaccines. Supasa et al. found a reduction of neutralization of vaccine sera and convalescent sera against B.1.1.7 variant (about 3.3 fold reduction for these vaccines) (236). Similarly, Zhou et al. found vaccine escape from their study. They found a decrease in vaccine sera against the B.1.351 variant (about a 7.6 fold reduction for these vaccines) (237).

### 7.2 Oxford-AstraZeneca Vaccine

It is a widely used vaccine and used by several countries like India, South Korea, etc. However, some studies found a reduction of the vaccine efficiency against the variants. Supasa et al. found a reduction of efficacy of Oxford-AstraZeneca vaccine against B.1.1.7 variant (about 3.3 fold reduction for these vaccines) (236). Similarly, Zhou et al. found a reduction of efficacy of Oxford-AstraZeneca vaccine against B.1.351 variant (about nine-fold reduction for this vaccine) (237).

### 7.3 BBIBP-CorV Vaccine and ZF2001 Vaccine

These two vaccines are from Chinese companies and are being used in China and Uzbekistan for vaccination. Huang et al. found a reduction of vaccine efficiency against the 501Y.V2 variant for this vaccine. They found a possible reduction of 1·5 to 1·6 fold in neutralizing geometric mean titers (238).

### 7.4 Moderna mRNA Vaccine (mRNA-1273) and Johnson & Johnson Vaccine (Janssen)

Moderna mRNA vaccine is being widely used in several countries like the USA, Canada, Europe, and several countries in the Middle East (239–242). The neutralization efficiency of the Moderna mRNA vaccine was observed similar to the BioNTech/Pfizer mRNA vaccine. However, reduced efficacy was noted for the Moderna mRNA vaccine due to the different mutations in the SARS-CoV-2 variants. Wang et al. observed that variants with the mutations E484K, K417N, N501Y, and E484K in the S-glycoprotein might reduce the vaccine’s efficiency. Other studies too noted the vaccine escape phenomena in the case of the Moderna mRNA vaccine (231). Finally, the scientists suggest that the mRNA vaccines may need to be restructured from time to time to end the vaccine evade phenomena (235). In another study, Garcia-Beltran et al. developed pseudoviruses with different RBD mutations or other parts of S-glycoprotein such as N501Y, K417N/T, and E484K, to illustrate the neutralization event. They found that five out of the ten pseudoviruses with mutations are distinctly resistant to neutralization (243). Carreño et al. also found a reduction in neutralization events from low to high against different variants (244).

Similarly, several researchers noted that the different variants reduced the neutralization efficiency of the Johnson & Johnson vaccine. Cele et al. pointed out the reduced activity by the variants, especially (501Y.V2) from country to country (57% in South Africa and 72% in USA) (227). Therefore, scientists recommend improving vaccines from time to time, considering the mutation and variants to avoid the vaccine escape.

## 8 The Strategy and Insight in Vaccine Design, mAbs Development, and Small Molecules (Chemical Components) Based Therapeutic Development and Their Escape

Most of the vaccine developed so far has considered the S-glycoprotein of Wuhan variants. Therefore, S-glycoprotein is the central point of vaccine development against SARS-CoV-2 (245). Researchers considered S-glycoprotein for vaccine development due to the highest antigenicity of the S-glycoprotein compared to other structural and non-structural proteins of SARS-CoV-2 (246). It has been noted that different types of vaccine platforms have been utilized for the vaccine development against COVID-19, such as an inactivated vaccine, subunit vaccines, nucleic acid vaccines, DNA vaccines, mRNA vaccines, recombinant vaccines, peptide and protein vaccine, VLPs (Virus-like particles), etc. (242, 247–249). Several countries approved these vaccines, and billions of people were vaccinated using them (1).

At the beginning of the pandemic, scientists have used immunoinformatics for the vaccine development for COVID-19, and several vaccine constructs were developed successfully (250–254). Due to the emerging SARS-CoV-2 variants and highly mutant variants, now the antigenicity of the epitope is altering. Therefore, it is a win-win situation for both the virus and the host. In this direction, vaccine partial escape events are noteworthy by the variants (231). Scientists suggest improving the development of the next-generation vaccine for the upcoming variants. In this direction, Bhattacharya et al. proposed a vaccine construct using alternative epitopes to fight against all newly emerging SARS-CoV-2 variants (255).

It has been observed that most of the antibody binds the RBD of S-glycoprotein to neutralize the antigenicity of SARS-CoV-2. Several studies have been performed to understand the interaction properties and the site between the neutralizing antibodies that bind the RBD of S-glycoprotein (256). Studies have tried to illustrate the neutralizing antibody structures and their interaction mechanism with epitopes to develop antibody-based therapeutic strategies (129, 257). However, several new SARS-CoV-2 variants have been generated with unique mutations in due course of time. The emerging variants of SARS-CoV-2 created a concern for antibody escape events (258). The preliminary study suggests that more mutations are localized in the antibody binding regions of S-glycoprotein. Therefore, scientists are trying to develop new strategies for nAb development. At the same time, to prevent the antibody escape, single antibody domains may be the ‘therapeutic choice’ against the SARS-CoV-2 variants. A combination of antibody domains may stop the emergence of SARS-CoV-2 variant from the antibody escape (222).

Several small molecule-based therapeutics have been approved for the treatment of COVID-19. In these cases, therapeutic development strategies have mainly been used to repurpose drugs (247, 259–261). Several clinical trials have been performed from small-size to large-size depending on the number of COVID-19 patients to understand the effectiveness of repurposing drugs. Some large clinical trials with most patients are ACTT-1, ACTT-2, and RECOVERY trials. These trials results showed the best therapeutic option, such as remdesivir or its combination with different antibodies (259). All the chemical components based therapeutic molecules have been developed using the drug targets, especially the primary drug target proteins of the virus or the host (262). However, the mutations in the drug target proteins were observed in the SARS-CoV-2 variants, and it is a significant concern today. One such example is remdesivir, which targets RdRp to inhibit the virus replication. Several mutations are reported in the RdRp in the newly emerging variants (258). It creates a concern for the chemical components based on therapeutic escape or therapeutic resistance.

## 9 T Cell Responses Against SARS-CoV-2 Variants

Successful T cell response can halt the spread of the virus (263). It has been noted that several viral variants might evade effective immune recognition and thereby T cell response. Specifically, it was pointed out that the variant can escape the CD8+ T cell. It is not a common phenomenon not only for SARS-CoV-2 variants; but also for other virus variants (264). After vaccination against SARS-CoV-2, T cell-mediated immunity obstructs the virus. Some studies noted that some variants do not invade the T cell-mediated immunity. A recent survey by Geers et al. observed that variants like B.1.351, B.1.1.7 do not invade the T cell-mediated immunity after BNT162b2 vaccination (265). However, after the emergence of the Omicron variant, new studies would be required to understand the T cell responses against the Omicron variant, as several people have contracted this variant even after being fully vaccinated.

## 10 Arrival of Immensely Mutated Omicron Variant: Is It a Concern for Antibody Escape as Well as Vaccine Escape?

A recent SARS-CoV-2 variant was noted at the end of November 2021 called Omicron. Scientists have observed nearly 50 mutations in the genome and about 32 mutations in the S-glycoprotein of this variant (266, 267). Omicron has several common mutations of the Alpha variant and Delta variant. The N501Y mutation is noted in the Omicron along with other variants. This mutation augments the S-glycoprotein affinity to hACE‐2 receptors, enhancing SARS-CoV-2 attachment with the host cell (267, 268). At the same time, E484 mutation in RBD of the S-glycoprotein has been found in the Omicron variant. This mutation is related to immune escape (267, 269). The Omicron variant also has a D614G mutation. The mutation helps to change the conformation of RBD regions and helps the fusion efficiencies with hACE‐2 receptors, thus enhancing transmission and increasing the stability for the SARS-CoV-2 replication. It finally enhances infectivity (270). The D614G mutation has been reported as a positive selection (4). Simultaneously, some mutations such as R203K and G204R are too noted in the Omicron variant. These mutations augment the adaptability of the virus’s evolution (267). Many other mutations have been found in the Omicron variant, such as Q493R, G496S, Q498R in RBD and throughout the S-glycoprotein. The role of many more mutations has not been reported so far. Thus, it is an urgent need to decipher the function of these mutations. Because of many mutations, the Omicron variant is rapidly spreading and replacing the Delta variant (271). Before two months ago, the Delta variant was the most dominating SARS-CoV-2 variant (272). At the same time, all the significant neutralizing antibodies escape phenomenon have been reported by the variant which has the capacity of evading the humoral immune response (273). Because of this, the variant is a concern for all the recent vaccines and, thereby, the vaccine effectiveness (274). Scientists are worried about the probable vaccine escape by this variant and are waiting for intensive research data. The emergence of Omicron has initiated a vaccine efficacy debate (275). Several scientists suggest booster doses of the vaccines for the fading immunity against the virus, while others favor rapid vaccine improvement to fight against the Omicron (276).

## 11 Limitations

The emergence of new SARS-CoV-2 variants has created a new direction of the pandemic. The emergence of new variants with new mutations creates a ‘fresh-thinking approach’ every time. After the evolution of the new SARS-CoV-2 variants, concerns regarding the vaccine escape and antibody escape event have been raised. One recent example is the identification of the Omicron variant. The quick spreading of the variant has forced scientists to consider vaccine improvement or new vaccine development for the variant. At the same time, scientists and policymakers are developing a new strategy to fight against the pandemic, for example, considering booster doses for fading immunity. Simultaneously, scientists are trying to illustrate the role of all the new mutations after the emergence of new mutations with unknown functions. It is highly desirable to know more about the new mutations and their functions in the developing variants. The review has not illustrated all these mutations due to the lack of understanding about all those new mutations.

## 12 Conclusion

The development of vaccines for COVID-19 is a unique success story in medical science and molecular biology, and it is an excellent example of collaboration and teamwork between researchers (277). The vaccine was rolled out within one year, and several billion vaccine doses have been administered worldwide (1). Now, emerging variants are developing throughout the globe, which is more challenging in this scenario. These variants are more prone to immune escape and partial vaccine escape. Both the escape process in light of the rising variant is enormously complex. There is clear evidence that the antigenicity changes due to the different variants, especially in the spike glycoprotein region, and the total phenomenon affects antibody neutralization. Presently it is a great challenge to understand the mechanism of immune escape and vaccine evasion. At the same time, we should appreciate the mutational process of the variants appropriately. It is also necessary to illustrate the details about the newly emerging variants generating from time to time and the efficiency of different vaccines escape against these variants. Moreover, an updated evaluation report is necessary regarding the vaccine’s efficacy against the new variants. There are still many unanswered questions regarding immune escape and partial vaccine escape of the emerging variants with escape mutations. These unsolved questions need to be resolved in the shortest time possible to end the pandemic and prepare for the new pandemic.

## Author Contributions

CC, conceptualization, investigation, writing- original draft preparation, reviewing and editing, and supervision. AS, validation, visualization, formal analysis, reviewing, editing, and funding acquisition. MB, validation, formal analysis, visualization. S-SL, review and funding acquisition. All authors contributed to the article and approved the submitted version.

## Funding

This study was supported by Hallym University Research Fund and by Basic Science Research Program through the National Research Foundation of Korea (NRF) funded by the Ministry of Education (NRF - 2020R1C1C1008694 and NRF- 2020R1I1A3074575).

## Conflict of Interest

The authors declare that the research was conducted in the absence of any commercial or financial relationships that could be construed as a potential conflict of interest.

## Publisher’s Note

All claims expressed in this article are solely those of the authors and do not necessarily represent those of their affiliated organizations, or those of the publisher, the editors and the reviewers. Any product that may be evaluated in this article, or claim that may be made by its manufacturer, is not guaranteed or endorsed by the publisher.
